# Axial compression performance of square concrete filled double skin SHS steel tubular columns confined by CFRP

**DOI:** 10.1038/s41598-023-37101-4

**Published:** 2023-06-21

**Authors:** Caisen Wang, Jiongfeng Liang, Wei Li, Chunfeng He

**Affiliations:** 1 Faculty of Civil & Architecture Engineering, East China University of Technology, Nanchang, China; 2 Key Laboratory for Structural Engineering and Disaster Prevention of Fujian Province, Huaqiao University, Xiamen, China; 3 College of Civil and Architecture Engineering, Wenzhou University, Wenzhou, China

**Keywords:** Engineering, Materials science

## Abstract

This paper focuses on the axial compression performance of 15 concrete-filled double skinned tubes CFDST columns with different CFRP reinforcement schemes. The design of this test used an outer square steel tube with a square steel tube inside, with concrete poured at the sandwich and the inner steel tube kept hollow. The structure is both cost effective and allows the hollow to be used for utility access. However, in recent years damage to CFDST has occurred due to fire, earthquakes, corrosion etc. Therefore, research into the reinforcement and repair of this structure is crucial. Compared to other reinforcement methods, FRP has the advantage of being lighter and more robust and does not significantly alter the original structure. In this study, the mechanical properties of the specimens were further analyzed from the data of load displacement, peak load and ultimate displacement by mainly observing and analyzing the damage mechanism of the specimens through the strengthening effect of different strengthening schemes for different hollow ratios. The results show that when the hollow ratio is not bigger than 0.33, the CFRP reinforcement effect is relatively obvious, especially the three-layer CFRP wrapped CFDST specimens have a substantial increase in bearing capacity and stiffness. Finally, an analytical study was carried out based on previous research and the experimental results agreed well with the calculated results.

## Introduction

Concrete filled steel tube (CFST) is a kind of composite structure of steel tube and concrete that offer superior strength, ductility, stiffness and energy absorption over ordinary steel, concrete or reinforced concrete structures. Due to the fully encapsulated nature of CFST structures, they have the advantage of reducing reinforcement rates and preventing concrete detachment compared to reinforced concrete with horizontally oriented hoops^1^. Earlier studies on CFST were carried out on varying the parameters of external steel tubes, particularly square steel tube concrete columns, and some linear regression models were proposed to predict describe axial compression damage^2,3^. Fam et al.^4^ evaluated the strength and ductility of CFST by combined axial compression and cyclic loading and compared it with the code and found that the code is on the conservative side. Then, in terms of numerical studies, the 3D finite element model simulation and parametric study about concrete-filled steel tube CFST under pressure was carried out by Choi et al.^5^ and Javed et al.^6^. Experimental and numerical comparisons were performed and the results were a good match.

With the development of CFST research, a kind of CFST composite structure with concrete-filled double steel tube(CFDST) comes into view. The CFDST column offer similar structural advantages to CFST columns^7^. In addition, they are lighter, stronger, and perform better energy uptake capabilities. Zhao et al.^8^ and Han et al.^9^ started a study on hollow sandwich steel tube concrete columns (SHS outer and SHS inner). Tao et al.^10^ carried out to investigate the interaction between the outer steel tube and the core concrete and described it as the confinement factor (*ξ*). Ekmekyapar et al.^11^ conducted axial compression tests on 16 specimens to compare the effect of diameter-thickness ratio of internal and external steel tubes and concrete grade on the mechanical properties of CFDST. In addition, some scholars have studied the geometric parameters of the inner and outer tubes^12–14^.

Although the composite CFST and CFDST structure has the merits of better load-bearing capacity and ease of construction, it also has shortcomings. As the steel tube is in the outermost layer, the surface is easy to corrode causing the later maintenance cost to increase, and it is easy to occur external buckling under the action of axial pressure. Carbon fibre-reinforced polymer (CFRP) is extensively used in civil engineering due to their light weight, high strength and corrosion resistance^15^. Park et al.^16^ put forward an axial design formula for CFRP-confined CFST columns by constraining the restraint efficiency factor of concrete. Prabhu et al.^17^ and Sundarraja et al.^18^ investigated the applicability of CFRP sheet for enhancing CFST columns under compression by considering the effects of number of layers, width and spacing. Al-Rousan et al.^19^ and Li et al.^20^ used FRP to enhance the corrosion and sulfate resistance of CFST. Liang et al.^21^ investigated the effect of different CFRP reinforcement schemes on the mechanical properties of recycled aggregate concrete filled steel tube (RACFT) columns. All of the above studies have shown good results for the reinforcement of CFST by FRP.

According to the study of CFRP confined normal CFST, if CFRP reinforcement technology is used on CFDST columns, the affection of extending the service life of the member with anticorrosive outer steel tube and higher ductility and energy absorption can be achieved^22–24^. Therefore, Talaeitaba et al.^25^ investigated the performance of FRP-reinforced CFDST at different length-to-diameter ratios and concrete strengths by means of finite element scaling. Skaria et al.^26^ has studied the effect of the axial behaviour of CFDST columns by modeling the species and thickness of FRP. Alias et al.^27^ carried out eccentric compression tests on FRP-reinforced CFDST columns and suggested that the load capacity increases with increasing restraint of the FRP. More recently, Wan et al.^28^ have investigated the effects of CFRP wrapped reinforced CFDST(SHS and CHS).

**Figure 1:**
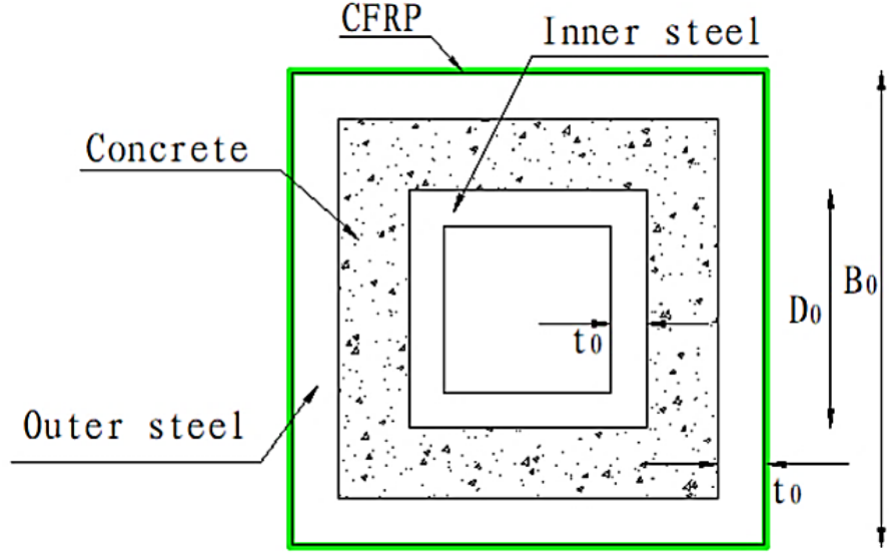
Cross-section of CFRP-confined CFDST stub column.

The damage to CFDST is caused by factors such as ageing, corrosion and fire, while FRP has the advantage of being lightweight and high strength. Based on the previous studies mentioned above, it is found that the research related to FRP-reinforced CFDST is relatively few and scattered, mainly focusing on numerical simulation studies, especially in terms of experimental research is not systematic. Therefore, in this report, an experimental study of CFRP-reinforced CFDST columns was carried out. Although a recent study of CFRP-reinforced CFDST(SHS and CHS) is to be just conducted by Wan et al.^28^ However, as square beam-column joints are generally easier to install and more widely used than circular joints, the cross-sections designed for this experiment are the outer SHS and the inner SHS as a supplement, such as the cross-section depicted in Fig. 1. In this paper, the mechanical behaviour of CFDSTs with different numbers of CFRP layers and hollow ratios is analyzed. In addition, an analytical study was carried out to more accurately predict the axial compressive strength of CFDST columns confined by CFRP sheet.

## Experimental investigation

### Material properties

The Q235 steel tube was used in this study the properties are shown in Table 1. In the design of the tests, the concrete strength was C30 and the water to cement ratio was 0.43. When the columns were made, a set of concrete specimens were reserved for the same batch based on the Code for the Design of Concrete Structures (GB50010-2010)^29^ and they had an average cubic compressive strength of approximately 36.3 MPa and modulus of elasticity of 30 GPa. As for CFRP, the properties and parameters of CFRP sheet are listed in Table 2.

**Table 1:** Properties of steel.

Type	Modulus (Gpa)	Yield strength (MPa)	Ultimate strength (MPa)	Ultimate strain (%)
Q235	206	230	337	39.8

**Table 2:** Properties and parameters of CFRP.

Type	${\text{Tensile stress }}P_{{\text{f}}} \quad {\text{(MPa)}}$	$Fracture \, strain\;\varepsilon_{FRP} \left( \% \right)$	${\text{Elastic}} modulus\;E_{FRP} \left( {{\text{GPa}}} \right)$	Width (mm)	Thickness (mm)
CFRP	3471	2.3	255	0.167	100

### Specimens design

A total of 15 CFDST column specimens were designed for this test, including 12 CFDSTs wrapped in CFRP sheet, 3 CFDST comparison specimens not wrapped in CFRP sheet. The tests were designed to explore the relevent mechanical properties of the specimens when axially compressed. Table 3 shows the detailed design parameters of the specimens.

**Table 3:** The parameters of specimens.

Specimen	Outer square steel tube B_0_ × t_0_ (mm)	Inner square steel tube D_0_ × t_0_ (mm)	Hollow ratio χ	Concrete strength grade	Column height H (mm)	Number of CFRP layers
S120 × 3_20 × 3_0	120 × 3	20 × 3	0.17	C30	500	–
S120 × 3_40 × 3_0	120 × 3	40 × 3	0.33	C30	500	–
S120 × 3_60 × 3_0	120 × 3	60 × 3	0.50	C30	500	–
S120 × 3_20 × 3_1a	120 × 3	20 × 3	0.17	C30	500	1
S120 × 3_20 × 3_1b	120 × 3	20 × 3	0.17	C30	500	1
S120 × 3_20 × 3_3a	120 × 3	20 × 3	0.17	C30	500	3
S120 × 3_20 × 3_3b	120 × 3	20 × 3	0.17	C30	500	3
S120 × 3_40 × 3_1a	120 × 3	40 × 3	0.33	C30	500	1
S120 × 3_40 × 3_1b	120 × 3	40 × 3	0.33	C30	500	1
S120 × 3_40 × 3_3a	120 × 3	40 × 3	0.33	C30	500	3
S120 × 3_40 × 3_3b	120 × 3	40 × 3	0.33	C30	500	3
S120 × 3_60 × 3_1a	120 × 3	60 × 3	0.50	C30	500	1
S120 × 3_60 × 3_1b	120 × 3	60 × 3	0.50	C30	500	1
S120 × 3_60 × 3_3a	120 × 3	60 × 3	0.50	C30	500	3
S120 × 3_60 × 3_3b	120 × 3	60 × 3	0.50	C30	500	3

B_0_ is the side length of the outer rectangular steel tube, D_0_ is the side length of the inner rectangular steel tube, t_0_ is the thickness of the pipe.

S120 × 3_20 × 3_1b: “S”—square steel tube;“120 × 3_20 × 3”—parameter of outer and inner steel tube;“1”—number of CFRP layers; “b”—distinguishing the same parameters.

### Specimens fabrication

In accordance with the design parameters in section "Specimens design", the inner and outer steel tubes are cut to the design length, ensuring that the two ends are flat and perpendicular to their axes. The end plates at both ends of the specimens are made of 150 mm × 150 mm, 10 mm thick steel plates. Position and weld the inner and outer tubes on the end plates, weld inside and then outside, use angle ruler to position, use short steel bars to position when welding the upper end to ensure concentric inside and outside. When pouring, mix the concrete and pour it through the gap between the inner and outer steel tubes, vibrating while pouring.

After maintenance, the top of the specimen is ground flush with a grinder and the upper end plate is welded. After removing the surface rust, the CFRP cloth is cut according to the length of the parcel, ZN-700 epoxy resin adhesive (AB glue) is prepared according to 2:1and positioned on the surface of the steel tube. The CFRP cloth is scraped and pressed to remove air bubbles and wrapped clockwise around horizontally. For multiple layers of CFRP, repeat the above steps when the previous layer is dry, with a lap length of 150 mm. Then leave for 5–7 days for all adhesive to set.

### Experimental set-up

**Figure 2:**
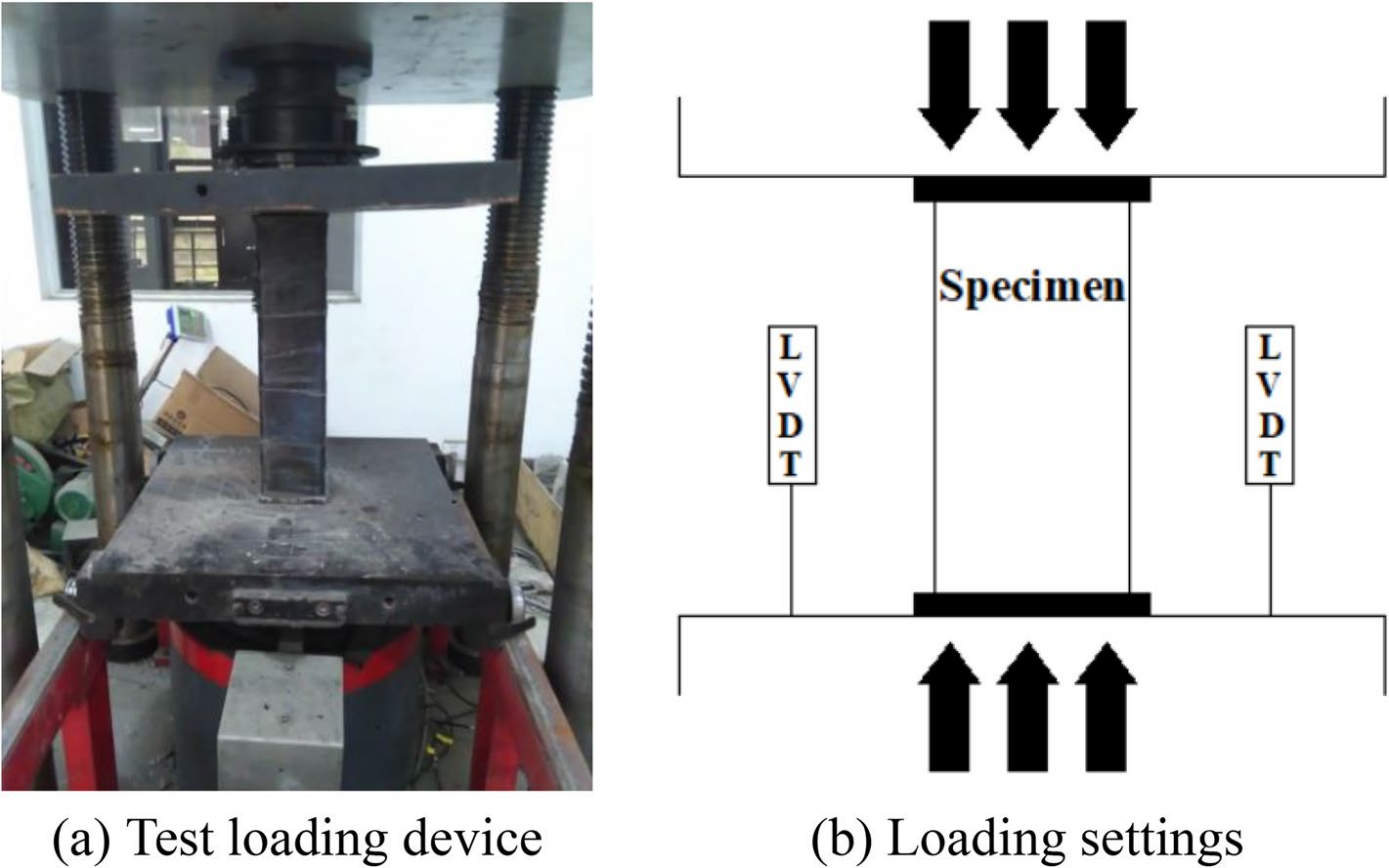
Test loading setup of columns.

The loading device used for this test is a YAW-3000 microcomputer-controlled electro-hydraulic servo press, which maximum range is 3000 kN. The experimental settings and equipment are illustrated in Fig. 2a. In the process of testing, the acquisition apparatus actively collects the test data of columns. From the Fig. 2b, two LVDTs were placed symmetrically on both sides of the specimen to measure the axial displacement. The specimens were manufactured with end plates welded on the top and bottom, and the top and bottom pressure plates were released directly from the ends of the specimens during axial pressurization. Before loading, preloading was carried out in the elastic range. The test was carried out in a graded manner, with each stage of loading being 1/10 of the estimated ultimate load in the elastic phase, and the next stage of loading was carried out for 2 min. When 0.6 times the predicted ultimate load is reached, the loading is reduced to 1/20 of the predicted ultimate load at each level and when damage is approaching, the loading is continued slowly until the specimen is damaged.

## Experimental results

### Peak loads and stiffness

The capability of CFDST (SHS outer and SHS inner) columns confined by CFRP sheets to resist elastic deformation under axial compression loading is discussed and the test results for all specimens are showed in Table 4. Where the peak load (*N*_u_) is defined as the ultimate strength, 60% of the peak load on the linear phase is defined as the yield load (*N*_y_), and the stiffness *E* as

$$E = \frac{{N_{{\text{y}}} }}{{\Delta_{y} }}$$

**Table 4:** Test results of the specimens.

Specimen	*N*_u_ (kN)	*N*_y_ (kN)	*Δ*_u_ (mm)	*Δ*_y_ (mm)	*E*_0_ (kN/mm)	*P*_1_ (%)	*P*_2_ (%)
S120 × 3_20 × 3_0	706	424	13	7.735	54.76	–	–
S120 × 3_20 × 3_1a	799	445	13	6.812	70.38	13.17	28.51
S120 × 3_20 × 3_1b	825	451	19	7.064	70.07	16.86	27.96
S120 × 3_20 × 3_3a	975	481	13.5	7.364	79.44	38.10	45.06
S120 × 3_20 × 3_3b	945	494	13.5	7.259	78.11	33.85	42.63
S120 × 3_40 × 3_0	763	458	14	7.572	60.46	–	–
S120 × 3_40 × 3_1a	842	491	12.5	7.12	70.96	10.35	17.36
S120 × 3_40 × 3_1b	882	497	10.5	6.959	76.05	15.60	25.78
S120 × 3_40 × 3_3a	990	535	13.5	6.907	86.00	29.75	42.24
S120 × 3_40 × 3_3b	1008	545	11.5	7.242	83.51	32.11	38.13
S120 × 3_60 × 3_0	718	431	13.5	5.618	76.68	–	–
S120 × 3_60 × 3_1a	765	440	11	5.135	89.39	6.55	16.57
S120 × 3_60 × 3_1b	779	448	13.5	5.236	89.27	8.50	16.41
S120 × 3_60 × 3_3a	868	487	11.5	4.577	113.79	20.89	48.39
S120 × 3_60 × 3_3b	884	478	12.5	5.034	105.36	23.12	37.40

The data in Table 3 present varying degrees of increase in stiffness for all CFDSTs with CFRP reinforcement. This is due to the CFRP circumferential wrapping confining the flexural deformation of the steel tube, or the existence of an interval between the circumferential strain elastic phase of the core concrete and the outer steel tube when compressed and the CFRP sheet. The CFRP sheet confines the further circumferential flexural deformation of the outer steel tube, thus improving the stiffness of the specimens.

### Axial load–displacement relationship

**Figure 3:**
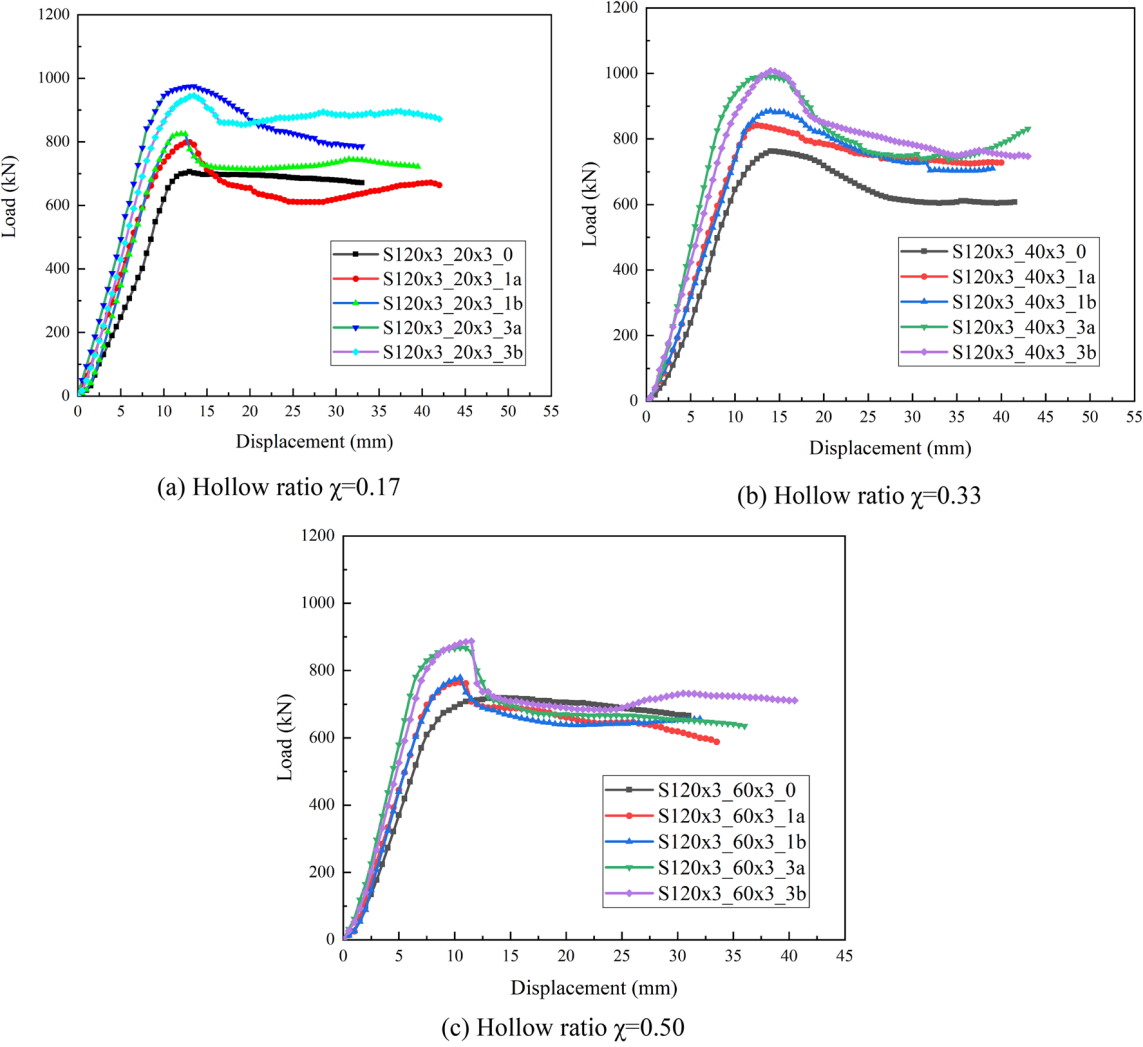
Load–displacement curves of tested specimens with different CFRP sheet layers.

**Figure 4:**
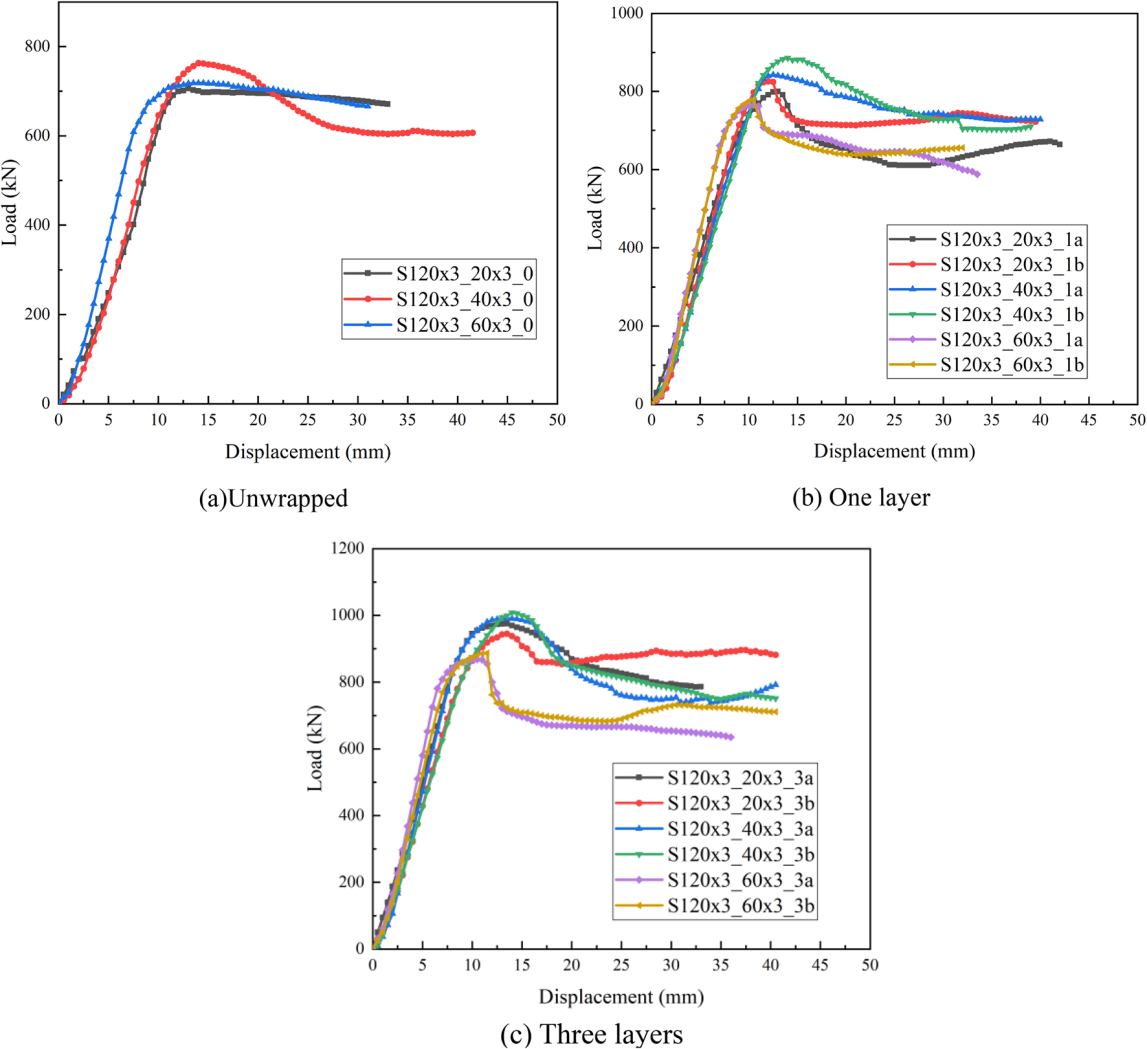
Load–displacement curves of tested specimens with different hollow ratio.

The load–displacement curves of axially compressed specimens for different CFRP layers and hollow ratios are illustrated in Figs. 3 and 4. The load–displacement curves of all test specimens contain three stages: elastic, elastic–plastic and plastic. As we can see from Fig. 4, at the beginning of loading, the tangents of the load–displacement curve which are normally larger, displacement increases slightly with external load and load–displacement is approximately linear; when a certain load is reached, the growth rate of the displacement becomes larger; When the axial load is continuously increased, there will be a point where the growth rate of the displacement starts to be significantly larger than the growth rate of the external load, until the specimen meets the ultimate load. For CFDST specimens, when the ultimate load is reached, the load changes little as the axial displacement increases after a short drop in the curve. For CFRP-reinforced CFDST specimen, the curve is similar to that of CFDST specimens at the beginning of loading. When the curve of CFDST specimen reaches the first peak point, the ratio suddenly decreases and the curve enters a different hardening stage from the initial stage, which is due to the presence of CFRP. In addition, as the number of CFRP layers increases, the load carrying capacity of the specimen increases more and more. When the CFRP fractures, the load carrying capacity of the CFRP-confined CFDST specimen suddenly decreases and the load displacement curve shows a steep drop, and after a short fluctuation, the trend of the curve is approximately the same as that of the CFDST specimen. The specimen has good ductility because its load carrying capacity does not show a trend of decreasing all the time even though a large plastic deformation occurs, but enters a long ductile phase. In addition, the more the number of CFRP layers, the larger the ultimate load capacity of the specimen and its corresponding axial displacement, indicating that CFRP layers increases the bearing capacity of the specimen and also increases the deformation capacity of the specimen.

Figure 4 depicts the impact of hollow ratio on the axial load–displacement curves of CFDST column specimens. Comparing the curves for the same CFRP reinforcement, it would be found that the velocity of the linear rise section grows with increasing hollow ratio. This indicates that the hollow ratio increases within a certain extent and with it the stiffness increases. Comparing the falling part of the curve after the peak load reveals that the speed of the load fall also follows the hollow ratio. In other words, a large hollow ratio falls faster and has a poorer deformation capacity than a smaller hollow ratio. Similarly, a comparison of the ultimate displacement reveals that it varies in the opposite trend to the hollow ratio.

### Discussion

#### The number of CFRP sheet layers

**Figure 5:**
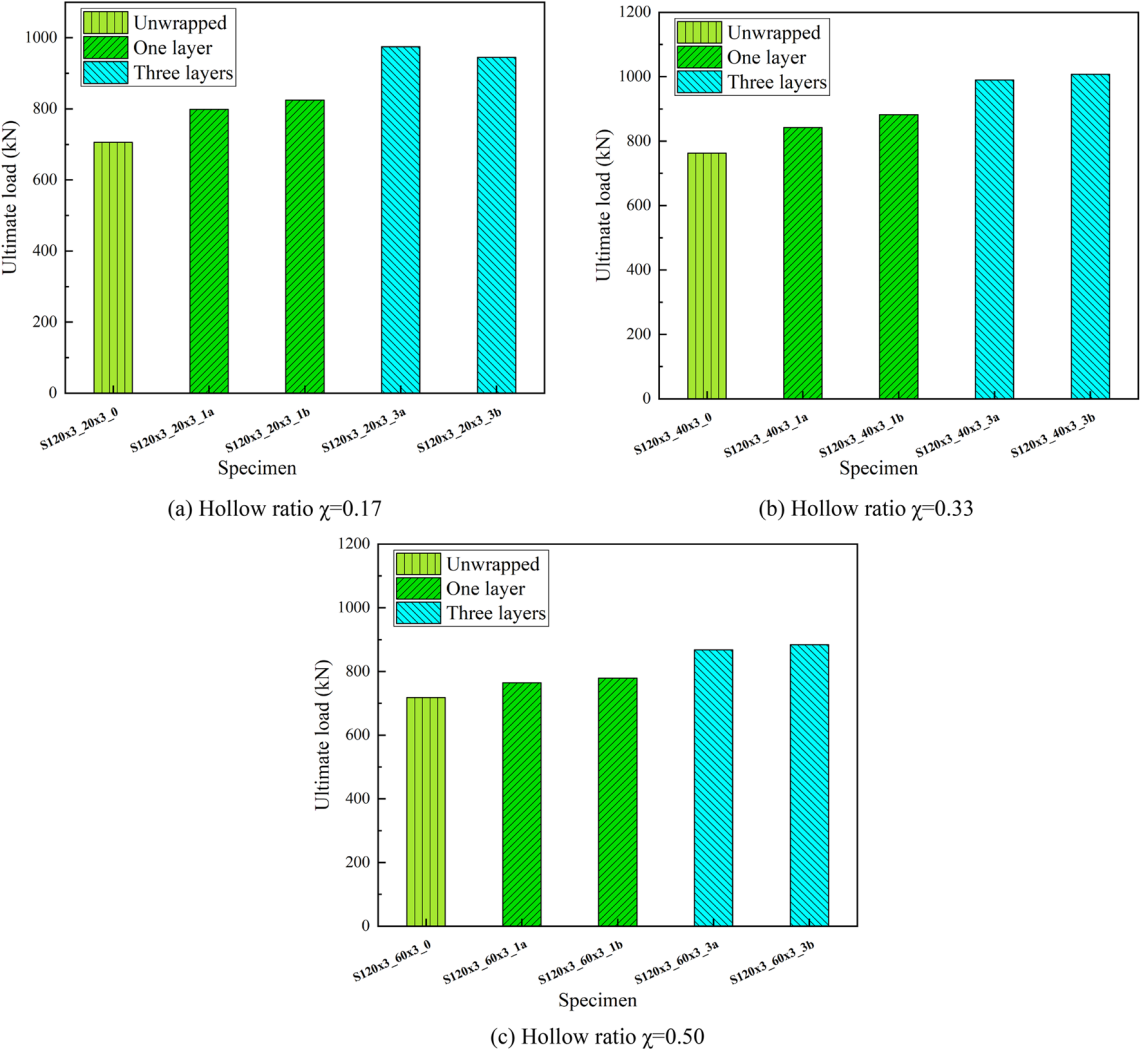
Effect of number of CFRP sheet layers on ultimate strength.

**Figure 6:**
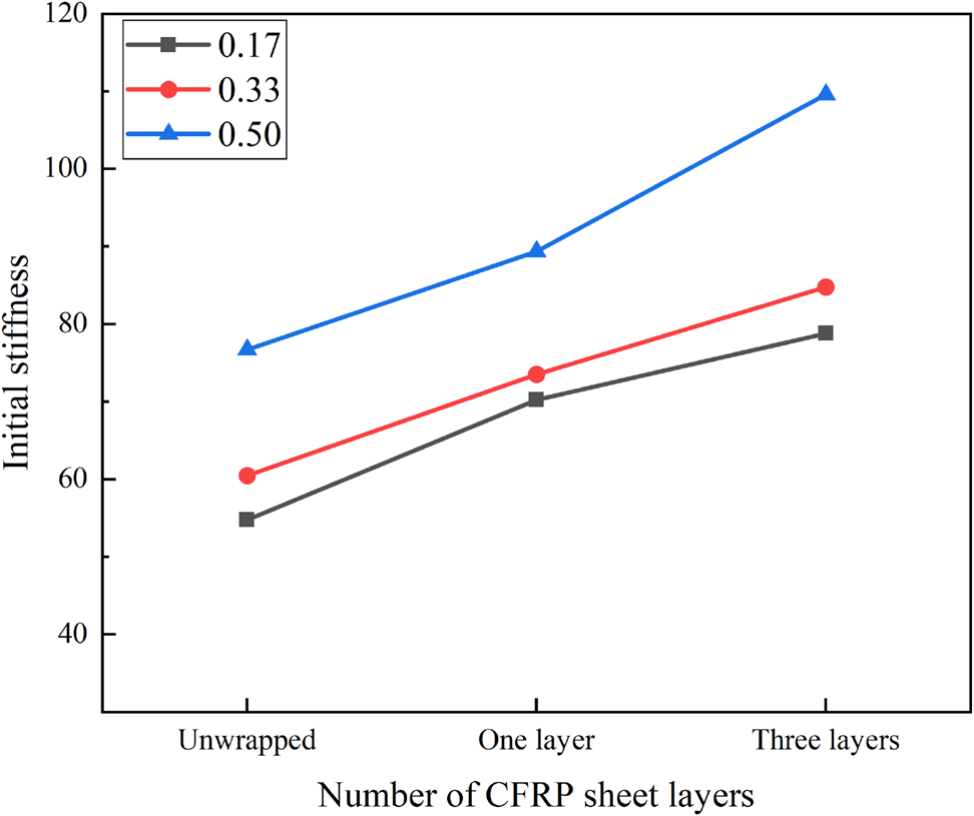
Effect of CFRP sheet layers on ultimate strength.

Figure 6 shows the ultimate strength as influenced by the number of layers of CFRP sheets. Obviously from Table 3 and Fig. 5, the CFDST columns wrapped in CFRP increase the ultimate strength to a greater extent. For the S120 × 3_20 × 3 series with a hollow ratio of 0.17, wrapping one layer of CFRP sheet and three layers of CFRP increased the ultimate strength by an average of 15% and 36%. For the S120 × 3_40 × 3 series, the average increase in ultimate strength is 13% and 31% for one and three layers respectively. Similarly, the ultimate strength of the S120 × 3_60 × 3 series specimens encased in one and three layers of CFRP with a hollow ratio of 0.50 increased by an average of 10.8% and 22% in varying degrees.

Figure 6 shows how the initial stiffness of specimens is influenced by the number of layers of CFRP sheet. It is clear that the resistance of the specimen to deformation is also enhanced due to the confinement of the CFRP sheet. At the hollow ratio of 0.17, the stiffness of the S120 × 3_20 × 3 series CFRP wrapped members was increased by 28.51%, 27.96%, 45.06% and 42.63% respectively compared to the unwrapped one. At the hollow ratio of 0.33 (S120 × 3_40 × 3 series), the degree of improvement is similar to that of the S120 × 3_20 × 3 series, with increases of 17.36%, 25.78%, 42.24% and 38.13% respectively. In contrast, the S120 × 3_60 × 3 series with a hollow ratio of 0.50, wrapped with one and three layers of CFRP, improved the stiffness of the members to a lesser extent, 16.57%, 16.41% and 48.39%, 37.4% respectively. It can be seen that the effect of wrapping one layer on stiffness varies for different hollow ratios.

From these results it is clear that the inner and outer steel tubes bear the axial loads and restrain the concrete according to their axial stiffness. For the core concrete, it bears most of the axial compression load and prevents inward local buckling of the steel tube. the CFRP covering delays the onset of outward local buckling of the steel tube and increases the restraint of the concrete. In the elastic phase the specimen bearing capacity and stiffness are well improved due to the interval between the CFRP and the circumferential strain of the steel tube and core concrete. In the later stages, as the load increases, the CFRP works in concert with it until the CFRP has failed.

#### Hollow ratio

**Figure 7:**
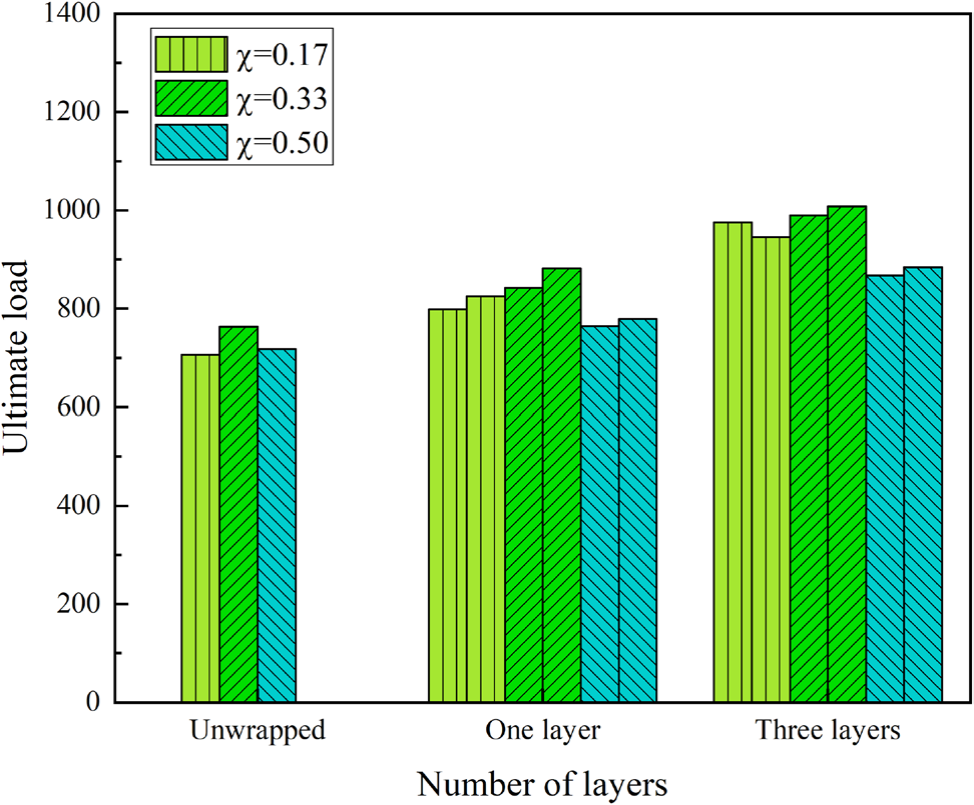
Effect of hollow ratio on ultimate strength.

Figure 7 shows the influence of hollow ratio to the ultimate strength of CFDST columns. It is obviously from the graph that the overall trend of the influence of hollow ratio to the ultimate strength of the CFDST columns are negatively correlated, with or without wrapping the CFRP sheet. However, as the hollow ratio changes from 0.17 to 0.33, the ultimate strength is increased. Specifically, the specimens with a hollow ratio of 0.50 unwrapped CFRP sheet was 5.9% lower than column S120 × 3_40 × 3_0 in terms of ultimate strength, which was similar to column S120 × 3_20 × 3_0. Analyzing the columns wrapped in one layer of CFRP sheet, the specimens with the hollow ratio of 0.33 had approximately 6.2% higher ultimate strength than the hollow ratio of 0.17, whereas the ultimate strength decreased by approximately 10.4% when the ratio increased to 0.50, similar to the specimen wrapped in three layers of CFRP sheet. As we can see the hollow ratio of 0.33 provides some improvement in the ultimate strength of CFDST columns.

**Figure 8:**
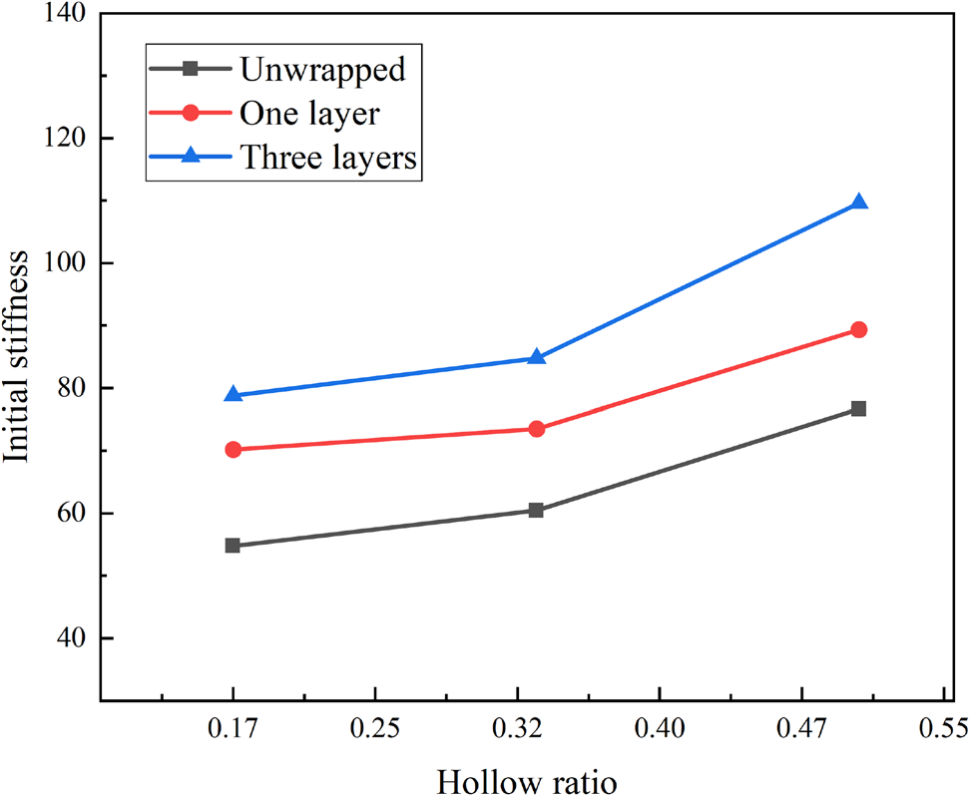
Effect of hollow ratio on initial stiffness.

Figure 8 illustrates the influence of hollow ratio on the initial stiffness to the columns. It is evident that the initial stiffness of the CFDST columns can be seen to be positively correlated with the hollow ratio. The higher the number of wrapped layers, the higher the initial stiffness growth rate. When wrapping one layer of CFRP sheet, the specimens with a hollow ratio of 0.50 increased by 21.5% compared to those with a hollow ratio of 0.33 and by 27.2% compared to those with a hollow ratio of 0.17. When wrapped in three layers, the members with a hollow rate of 0.50 improved by 29.3% and 39.1% over those with a hollow rate of 0.33 and 0.17 respectively. Similarly, for unwrapped specimens the improvement was 26.8% and 40% respectively.

### Failure modes

#### CFDST confined by CFRP

**Figure 9:**
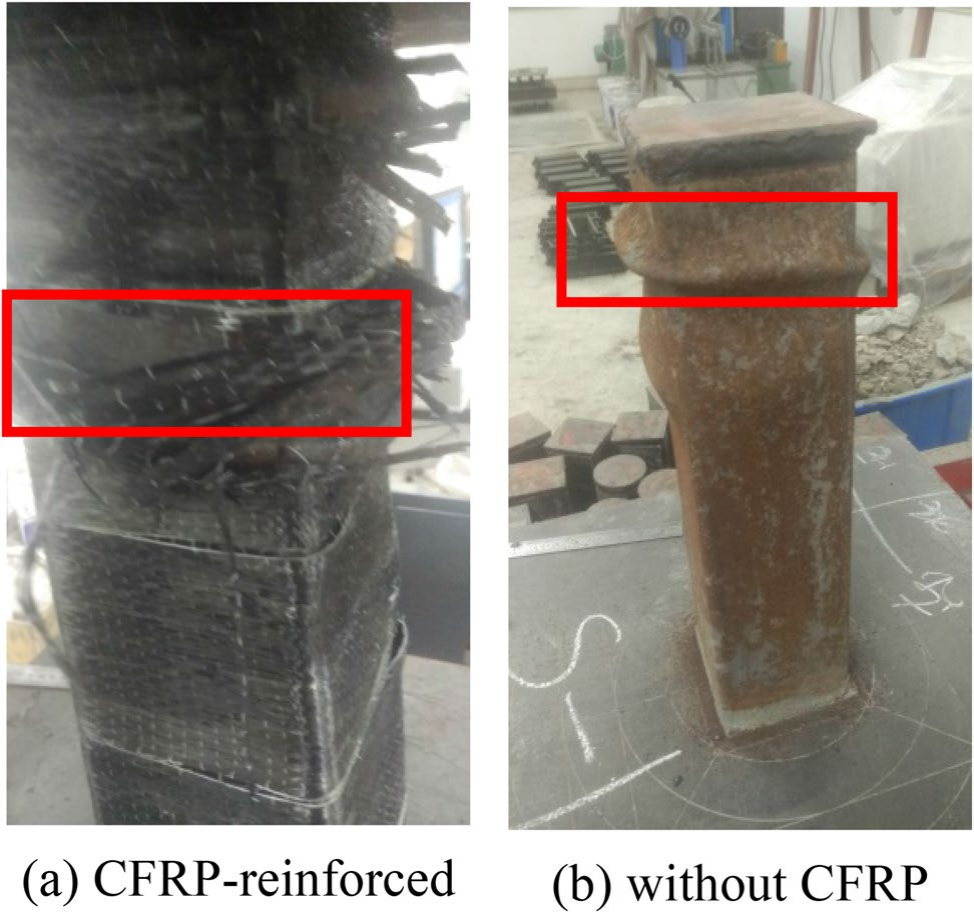
Typical failure modes of the tested columns.

At the early stage of loading, the axial pressure was approximately proportional to the longitudinal strain, and there was no visible phenomenon on the specimens surface. As the load increased, the epoxy resin shedding sound could be heard sporadically due to the outer CFRP sheet. When the load is constantly increasing, local convexity starts to appear in the middle of the specimen and its nearby area. When the load increased to 90% of *N*_max_, the specimen showed obvious deformation and the CFRP sheet started to fracture in small amount from the right angle corner. As the load continues to increase, a large amount of CFRP fractures, which develops approximately uniformly from the middle to the ends of the column, as shown in Fig. 9a.

#### CFDST without CFRP

However, with the same parameters for specimens without CFRP wrapping, no visible phenomena were seen on the specimens at the surface during the early stages of loading. In contrast, the localized bulging of the tubes appeared earlier. When the load was increased to about 70% of *N*_max_, the outer surface of the specimen started to bulge. With the further increase of the load, the phenomenon is more obvious and the deformation damage is relatively faster. The final damage showed that the middle and both ends of the tube were deformed and flexed outward, similar to the elephant foot-shaped damage, as shown in Fig. 9b.

The reason for these different phenomena lies mainly in the action of the CFRP. When the CFDST is subjected to axial compression, the plastic deformation of the inner and outer steel tubes is too large and the restraining effect on the core concrete decreases. For the CFRP-reinforced specimen, as the circumferential strain of the CFRP lags behind the circumferential strain of the outer steel tube, the CFRP sheet confines the deformation of the steel tube, forming a hoop effect that cooperates with the steel tube to confine the core concrete and improve the load bearing capacity of the specimens.

## Simplified formulate

### Design model of CFDST

According to GJB4142-2000 (2001)^30^ and study of Park^31^, the constraint effect of concrete, the so-called confinement factor *ξ*, is proposed, and the state of the outer steel tube and the concrete of the sandwich layer in CFDST is equivalent to the state of action of the steel tube and the core concrete in CFST columns. The CFDST column ultimate strength can be expressed as:

1 $$N_{u} = A_{sc} f_{scy} + A_{s2} f_{y2}$$

2 $$A_{{{\text{sc}}}} = A_{{\text{c}}} + A_{s1}$$

3 $$f_{scy} = (1.212 + B\zeta + C\zeta )f_{ck}$$

4 $$B = 0.1381f_{y1} /235 + 0.7646$$

5 $$C = - 0.0727f_{ck} /20 + 0.0216$$

6 $$\zeta = A_{s1} f_{y1} /A_{c0} f_{ck}^{{}} = \alpha_{0} f_{y1} /f_{ck}$$

7 $$\alpha_{0} = A_{s1} /A_{c0}$$

$f_{ck}$ is characteristic strength of concrete defined as $f_{ck} = 0.67f_{cu}$.Among them, *f*_scy_—the strength bearing capacity of the outer steel tube and the core concrete of axial compressive; *A*_sc_—the area of the outer steel tube and the core concrete; *f*_y1_—the yield strength of outer steel tube; *A*_s1_—area of outer steel tube; *f*_y2_—the inner steel tube yield strength; *A*_s2_—area of inner steel tube; *f*_ck_—standard value of concrete compressive strength; *f*_cu_—cubic compressive strength of the concrete; *A*_c0_—area surrounded by outer steel tube; *α*_0_—section nominal steel content; *ζ*—constraint effect coefficient.

### The model of CFDST confined by CFRP

**Figure 10:**
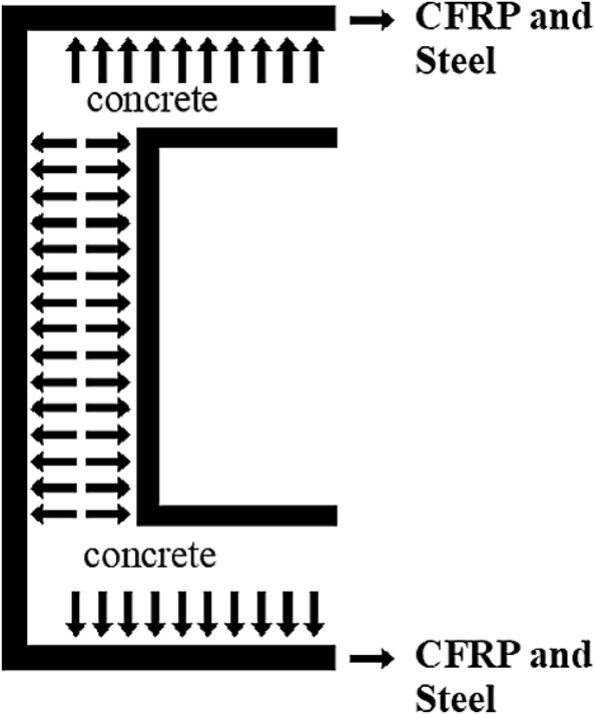
Confinement pressure provided by the CFRP.

The core concrete was subjected to confining pressures provided by the CFRP sheet and the steel tube, as shown in Fig. 10. This is because the restraint of the CFRP delays the buckling of the steel tube and increases the restraining effect of the pipe on the concrete to some extent. All specimens underwent deformation of the steel tube and buckling of the ends. In this study, the lateral confinement pressure (*f*_*l*_) of the FRP-reinforced CFDST column can be explained by Eq. (8) due to the fact that the specimens are confined by the steel tubes and the FRP sheets.

8 $$f_{l} = \frac{{2f_{s} t_{s} }}{d} + \frac{{2f_{FRP} t_{FRP} }}{d}$$

According to several now available strength models for FRP-steel-confined concrete, Park et al.^28^ take the following form

9 $$\frac{{f_{cc}^{^{\prime}} }}{{f_{co}^{^{\prime}} }} = 1 + k_{1} \frac{{f_{l} }}{{f_{co}^{^{\prime}} }}$$

However, based on the studies of Xiao et al.^32^ and Tao et al.^33^, the result is expressed in Eq. (10).

10 $$\frac{{f_{cc}^{^{\prime}} }}{{f_{co}^{^{\prime}} }} = 1.56 + 2.13\frac{{f_{l} }}{{f_{co}^{^{\prime}} }}$$

So the ultimate capacity (*N*_u_) is demonstrated

11 $$N_{u} = (A_{c} + A_{s1} )f_{scy} + A_{s2} f_{y2}$$

where *t*_s_ = *t*_0_, *d* = *B*_0_, *f*_s_ = *f*_y1_, *t*_FRP_—width of specimens wrapped in CFRP; *f*_FRP_—tensile strength of FRP.

**Figure 11:**
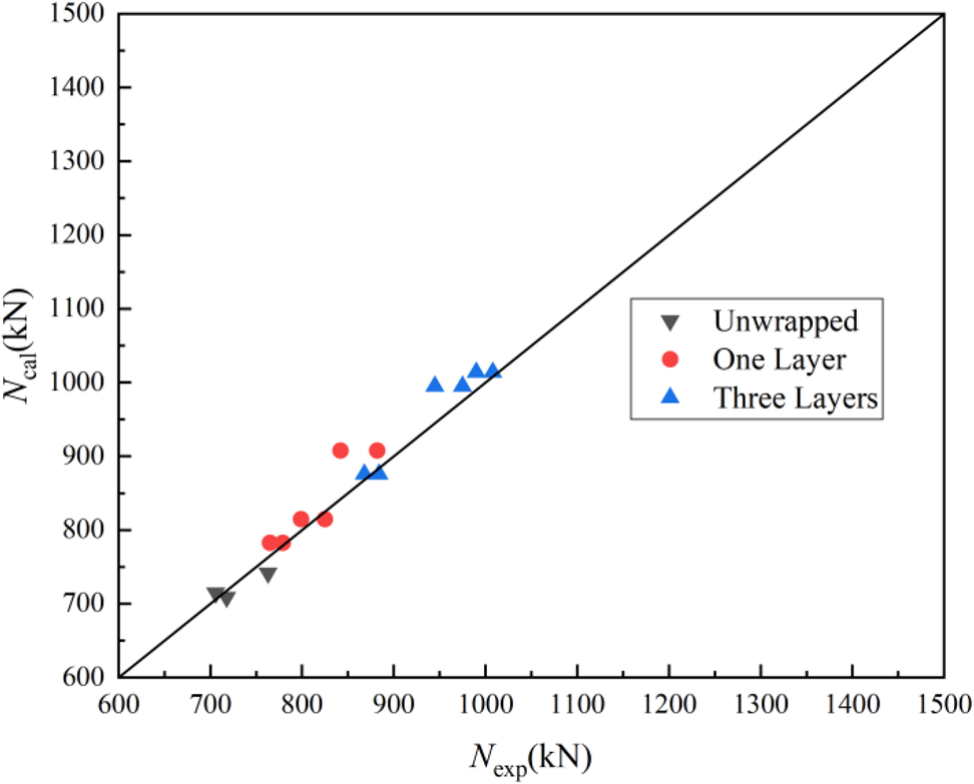
Comparison between experimental and calculated results.

According to Eq. (11) for CFDST confined by CFRP to calculate the bearing capacity *N*_cal_, the pair of test value and theoretical calculation value is shown in Fig. 11. It is found that Eq. (11) can accurately predict the ultimate bearing capacity of CFDST confined by CFRP, and the analytical study developed in this paper can provide reference for the engineering application to predict the ultimate bearing capacity of CFDST confined by FRP. Especially in CFDST structural repair and reinforcement.

## Conclusions

The mechanical properties of the CFRP-confined-CFDST (SHS outer and SHS inner) columns have been studied and the data analyzed in this paper. From the experimental results, we can draw the conclusions as following:

1. The axial bearing capacity and initial stiffness of the columns were positively correlated with the number of CFRP sheet layers. For the specimens with hollow ratio no more than 0.33, the bearing capacity and stiffness were significantly increased. Among them, the stiffness of CFDST wrapped with 1 layer of CFRP sheet generally increased by 20%. The CFDST wrapped with 3 layers of CFRP sheet generally increased the bearing capacity by 30%.
2. The synergistic effect of CFRP and outer steel tube restrains the buckling of the outer steel tube, especially in the end. Therefore, the CFDST with CFRP reinforcement fails as a fracture in the middle of the CFRP, while the inner steel tube is locally buckled at the middle height.
3. According to the load displacement curve, the lateral restraint of the core concrete is enhanced after the elastic stage due to the synergistic restraint effect of CFRP and outer steel tube to improve the bearing capacity. although the bearing capacity will drop suddenly after the CFRP fracture, the specimen shows a long ductile trend due to the strain hardening effect of the steel tube.
4. Based on the experimental results an analytical model was developed to predict the load carrying capacity of the CFRP-confined CFDST (SHS and SHS) column.
5. More different reinforcement schemes for CFRP need to be further investigated and the uneven distribution of the confining stresses along the radial direction due to the presence of the internal steel tubes.

## Data Availability

All data generated or analyzed during this study are included in this published article.
